# Cryoglobulinemia Complicated by Pneumonia

**DOI:** 10.7759/cureus.75466

**Published:** 2024-12-10

**Authors:** Luka Kiguradze, Elizabeth Onoprishvili, Khatuna Shvelidze, Kakha Vacharadze

**Affiliations:** 1 Internal Medicine, David Tvildiani Medical University, Tbilisi, GEO; 2 Internal Medicine, Israeli-Georgian Multiprofile Medical Center “Healthycore”, Tbilisi, GEO; 3 Pulmonology, Israeli-Georgian Multiprofile Medical Center “Healthycore”, Tbilisi, GEO

**Keywords:** autoimmune arthritis, cryoglobulinemia vasculitis, idiopathic cryoglobulinemia, interstitial pneumonia, secondary bacterial pneumonia

## Abstract

This study describes a 64-year-old female with a history of hepatitis C and cryoglobulinemia, who presented with respiratory symptoms, including dry cough, shortness of breath, and fever, alongside joint pain and fatigue. Initial workup revealed interstitial pneumonia, supported by chest imaging, and the patient was treated for pneumonia with standard antibiotic therapy. Despite no renal involvement, a hallmark of cryoglobulinemia, further testing confirmed elevated serum cryoglobulin levels. The patient was diagnosed with cryoglobulinemia complicated by pneumonia, a rare but significant manifestation of this disorder. Treatment was initiated with corticosteroids and immunosuppressive therapy.

Cryoglobulinemia typically presents with renal complications, such as membranoproliferative glomerulonephritis, but this case highlights an atypical involvement of pulmonary pathology and arthritis without renal dysfunction. The patient responded well to therapy and was discharged with outpatient follow-up. This report underscores the importance of considering cryoglobulinemia in the differential diagnosis of patients with vasculitis symptoms, even when renal manifestations are absent. Additionally, it highlights the limited documentation of the association between cryoglobulinemia and pulmonary complications. Noting this association is crucial so that a clearer pattern can be established if more cases arise.

## Introduction

Cryoglobulinemia is a rare disorder characterized by the presence of abnormal proteins called cryoglobulins in the blood [1]. These proteins precipitate at low temperatures and dissolve upon warming. Cryoglobulinemia can cause a variety of symptoms, including purpura, arthritis, and glomerulonephritis, due to the deposition of cryoglobulins in small- to medium-sized blood vessels [2,3].

Cryoglobulins are generally divided into three groups as follows: type I - monoclonal antibodies (immunoglobulin M or immunoglobulin G); type II - mixed polyclonal and monoclonal antibodies, such as monoclonal IgM with rheumatoid factor activity and polyclonal IgG; type III (most common) - polyclonal IgG and IgM antibodies [4-7]. This study discusses a patient with cryoglobulinemia complicated by pneumonia, which is a rare possible complication seen in cryoglobulinemia, illustrating the diagnostic and therapeutic challenges in such cases [8-12].

## Case presentation

Clinical presentation

A 64-year-old female presented with a chief complaint of a week-long history of dry cough. The patient also had shortness of breath, fatigue, joint pain, and 38°C fever. Over a week, these symptoms worsened, with the patient experiencing increased breathlessness and fatigue.

Clinical history

The patient had a medical history of type 2 diabetes mellitus, managed for five to six years with metformin/sitagliptin (50/1000 mg) twice daily, and gliclazide (60 mg) once daily; hypothyroidism treated with levothyroxine (125 µg); other medications include magnesium hydroxide + acetylsalicylic acid (75 mg) and atorvastatin (20 mg); and surgeries including partial thyroidectomy, hysterectomy with adnexectomy, and cholecystectomy. The patient was diagnosed with hepatitis C in 2009, which was treated with interferon and ribavirin, and experienced a bilateral, non-pruritic purpuric rash on the lower limbs in 2016, which was treated with glucocorticosteroids by a rheumatologist on suspicion of being an immune thrombocytopenic rash, with remission until March 2024. In March 2024, she experienced a recurrence of her symptoms which was treated with hormonal therapy, improving her condition, but purpura recurred after stopping medication once again. The patient also began experiencing periodic episodes of polyarthralgia for several months prior to presenting to the emergency room.

Physical examination

The patient was in distress and was admitted to the emergency room. Vital signs were as follows: heart rate of 115 beats per minute, blood pressure of 95/60 mmHg, sinus rhythm, respiratory rate of 34 breaths per minute, and oxygen saturation of 85%. Pulmonary examination revealed bilaterally decreased breath sounds with crepitus. Cardiological examination showed no murmurs or thrills. Neurological examination showed the patient to be adynamic, and lethargic, but no sensory-motor deficits. The abdominal examination revealed no tenderness or rebound tenderness and no signs of hepatosplenomegaly; the kidneys were not palpable and urine flow was uninterrupted. Skin examination noted no peripheral edema or vasculitis.

Imaging

**Figure 1:**
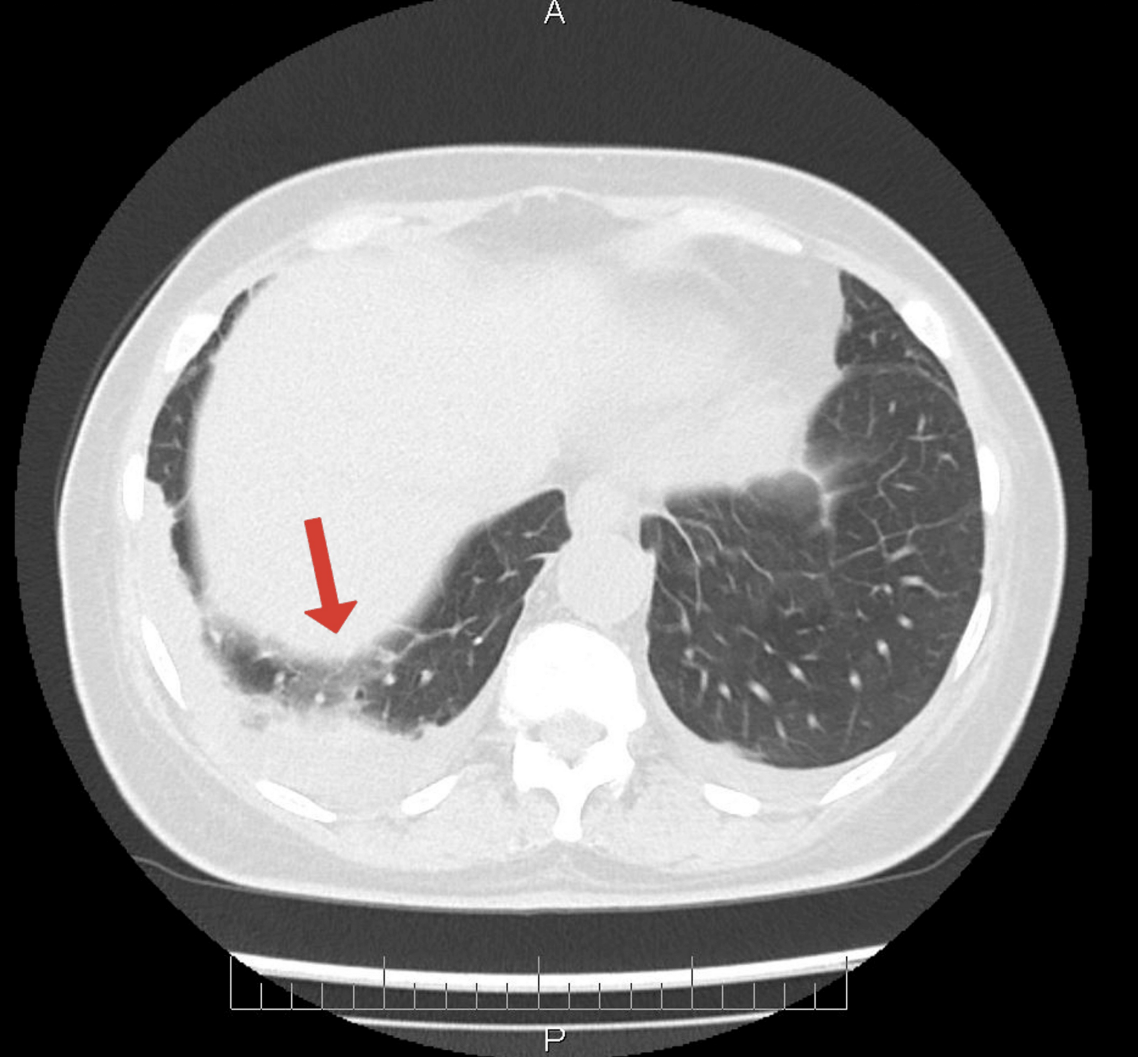
Non-contrast chest CT showing fibrotic changes and pneumonia. The arrow points out the most visible fibrotic changes and pleural effusion measuring 1.5 cm.

The chest X-ray showed opacification of both lower basal lung fields due to fluid, with no other changes. Chest computed tomography showed fibrotic changes, interstitial infiltrates, enlarged lymph nodes up to 1.0 cm in the mediastinum, bilateral dorsal and basal subpleural infiltrative changes, and small pleural effusions bilaterally up to 1.5 cm (Figure 1).

The patient was treated as an inpatient for pneumonia with a regimen of ceftriaxone and azithromycin and was also administered supplemental oxygen, in accordance with current standards of care. Given the patient's history of autoimmune disease and arthralgia, various laboratory tests were conducted to investigate the potential underlying cause of the patient's presentation (Table 1).

**Table 1:** Laboratory tests conducted during the patient's hospitalization.

Test	Result	Reference range
COVID-19 antigen PCR test	Negative	-
Complete blood count (CBC)		
White blood cells (WBC)	4.17 x 10/mm^3^	4500-11,000/mm^3^
Red blood cells (RBC)	3.99 x 10/mm^3^	3.5-5.5/mm^3^
Hemoglobin (HGB)	11.0 g/dL	12.0-16.0 g/dL
Hematocrit (HCT)	32.8%	36-46%
Mean corpuscular volume (MCV)	82.2 fL	80-100 fL
Platelets (PLT)	330 x 10^3^/μL	150,000-400,000/mm^3^
Erythrocyte sedimentation rate (ESR)	30 mm/h	0-20 mm/h
Coagulation studies		
Prothrombin time (PT)	11 s	11-15 s
Prothrombin index	85.6%	85-100%
International normalized ratio (INR)	1.08	0.8-1.1
Partial thromboplastin time (PTT)	24.2 s	25-40 s
Thrombin time	19.0 s	14-21 s
Fibrinogen	300 mg/dL	200-400 mg/dL
Creatinine	44.1 μmol/L	53-106 µmol/L
Blood group	O(I), Rh(-)	-
C-reactive protein (CRP)	25.9 mg/L	8-10 mg/L
Anti-cyclic citrullinated peptide (anti-CCP)	Negative	-
Rheumatoid factor	171.63 U/mL	<20 U/mL
Antinuclear antibody (ANA IgG)	Negative	-
pANCA	Negative	-
cANCA	Negative	-
Complement studies		
Complement factor C3	1.2 g/L	0.75-1.75 g/L
Complement factor C4	0.12 g/L	0.16-0.48 g/L
C1q	145 mg/L	127-201 mg/L
Other tests for systemic lupus erythematosus and related pathologies	Negative	-

Following negative test results for systemic lupus erythematosus and other related pathologies, mixed cryoglobulinemic vasculitis was suspected. Subsequent testing showed elevated serum cryoglobulin concentration and methylprednisolone 16 mg 2x per day, azathioprine 50 mg 1x per day, and esomeprazole 40 mg 1x per day therapy was initiated. The patient exhibited symptomatic improvement in both joint pain and respiratory function and was subsequently discharged to be managed as an outpatient.

## Discussion

This study highlights a rare presentation of pneumonia in a patient with cryoglobulinemia who notably does not have any renal involvement. Cryoglobulinemia typically presents with renal complications due to cryoglobulin deposition in the kidneys, often leading to membranoproliferative glomerulonephritis. However, in this patient, all renal function tests, including serum creatinine, blood urea nitrogen, and urinalysis, came back normal, indicating the absence of renal involvement.

A significant proportion of cryoglobulinemia cases are associated with hepatitis C virus (HCV) infection [13,14]. In one study, in 92% of cases, the presence of HCV infection was demonstrated [15]. The chronic infection triggers the immune system to produce cryoglobulins, leading to mixed cryoglobulinemia. This association highlights the importance of screening for HCV in patients presenting with cryoglobulinemia. Mixed cryoglobulinemia has been observed to be more common in Southern Europeans and women (a female-to-male ratio of 3:1), which fits in line with our patient’s presentation [16].

A similar case of pulmonary involvement in cryoglobulinemia is reported in a patient with long-standing essential mixed cryoglobulinemia (EMC) who developed interstitial and cavitary lung disease, histologically consistent with bronchiolitis obliterans organizing pneumonia (BOOP). The authors propose a potential etiologic link between connective tissue diseases, such as EMC, and the development of BOOP [17].

A thorough literature review was conducted using PubMed, searching for cases of pulmonary involvement in cryoglobulinemia in English, Spanish, French, and German. The relevant publications have been studied but such a presentation remains a rarity, emphasizing the uncommon nature of pulmonary manifestations in cryoglobulinemia without concurrent renal disease.

This unusual presentation emphasizes the need for clinicians to consider a broad differential diagnosis when evaluating patients with cryoglobulinemia and respiratory symptoms, even in the absence of renal involvement. The findings from this study contribute to the limited body of knowledge on the pulmonary complications of cryoglobulinemia and could aid in establishing a pattern, as more cases could potentially be reported in the future.

## Conclusions

This study highlights a rare case of cryoglobulinemia complicated by pneumonia without renal involvement. While cryoglobulinemia typically leads to renal issues such as membranoproliferative glomerulonephritis, our patient exhibited no renal abnormalities, as confirmed by normal renal function tests. This report contributes to the limited documentation of such presentations, emphasizing the necessity for clinicians to maintain a broad differential diagnosis when assessing vasculitis with signs of pulmonary disease. Recognizing this rare clinical manifestation can facilitate timely and appropriate management, thereby improving patient outcomes.
